# A Rare Case of a Solitary Fibrous Tumor of the Prostate: A Case Report

**DOI:** 10.7759/cureus.100720

**Published:** 2026-01-03

**Authors:** Sahar Kabani, Azizun Nisa, Zeeshan Uddin

**Affiliations:** 1 Pathology and Laboratory Medicine/Histopathology, Aga Khan University Hospital, Karachi, PAK

**Keywords:** cd34, malignant, prostate, solitary fibrous tumor, stat6

## Abstract

Spindle cell lesions of the prostate are rare and diagnostically challenging, encompassing entities such as stromal tumors of uncertain malignant potential (STUMP), sarcomas, sarcomatoid carcinoma, and mesenchymal tumors, including solitary fibrous tumor (SFT), which is exceedingly uncommon in the prostate.

A 64-year-old male presented with progressively worsening lower urinary tract symptoms over several months, culminating in an episode of acute urinary retention. The patient underwent transurethral resection of the prostate (TURP) for symptomatic relief and tissue diagnosis. TURP was performed due to persistent obstructive urinary symptoms and acute urinary retention. Histopathological examination revealed a cellular spindle cell neoplasm arranged in haphazard sheets and fascicles, associated with intervening staghorn-shaped vasculature and a hyalinized stroma. The tumor exhibited increased mitotic activity and an expansile, non-infiltrative growth pattern. Immunohistochemical analysis demonstrated strong nuclear STAT6 expression and diffuse CD34 positivity, while epithelial, myoid/myofibroblastic, neural, and synovial sarcoma markers were negative, confirming the diagnosis of SFT. Although the diagnosis was established based on morphology and immunoprofile, adverse histologic features, such as increased mitotic activity and high cellularity, suggest potential malignant behavior, warranting close clinical follow-up.

This case underscores the importance of thorough histopathological and immunohistochemical evaluation in distinguishing SFT from other spindle cell lesions of the prostate, particularly given its extreme rarity and potential implications for patient management and follow-up.

## Introduction

Spindle cell lesions of the prostate are rare and encompass prostate-specific tumors such as hyperplastic nodules of prostatic stroma, stromal tumors of uncertain malignant potential (STUMP), stromal sarcoma, and sarcomatoid carcinoma, as well as mesenchymal lesions, including solitary fibrous tumor (SFT), smooth muscle tumors, rhabdomyosarcoma, inflammatory myofibroblastic tumor, and prostatic synovial sarcoma. The prostate may also be secondarily involved by a gastrointestinal stromal tumor (GIST) extending from the adjacent large bowel. These lesions often present considerable diagnostic challenges for pathologists due to overlapping histomorphological features, and accurate distinction is essential, given the differences in prognosis and management among these entities [1-3].

SFT of the prostate is an exceedingly rare mesenchymal neoplasm, with 74 cases reported in the literature to date, according to the most recent systematic review [4]. Documentation has increased over the past decade, with improved recognition and the use of STAT6 immunohistochemistry. Most prostatic SFTs follow a benign course with a favorable prognosis; however, approximately 10% demonstrate malignant potential, characterized by increased cellularity, mitotic activity, and nuclear atypia, with potential for recurrence or metastasis. Awareness of these features is essential for appropriate management and follow-up [2,5,6].

In this report, we present a case of a 64-year-old male patient diagnosed with SFT of the prostate. This report contributes to the literature by detailing histopathological and immunohistochemical features that distinguish SFT from other spindle cell lesions.

## Case presentation

We received transurethral resection of the prostate (TURP) chips in our department for histopathological evaluation. As the patient had not been assessed in the urology clinics at our institution, only limited but relevant clinical information - including symptomatology, prior investigations, and therapeutic interventions - was subsequently obtained through telephonic correspondence with the patient’s attendant. The 64-year-old male, with no significant past medical or surgical history, reported moderate lower urinary tract symptoms and nocturia for the past year, along with a single episode of acute urinary retention, which prompted him to seek medical attention and undergo urological evaluation at a peripheral facility. Transrectal ultrasound and digital rectal examination both demonstrated prostatic enlargement.

**Figure 1:**
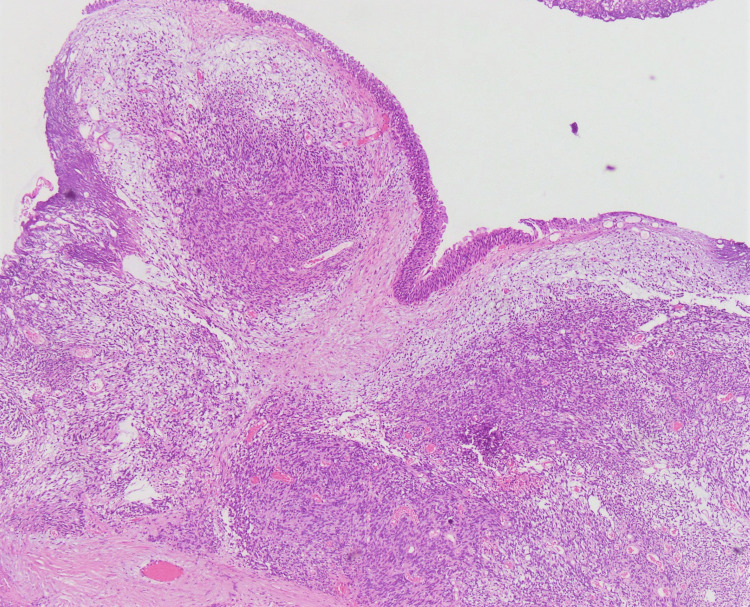
Prostatic tissue, displaying overlying urothelium, shows involvement by a tumor composed of spindle-shaped cells arranged in a haphazard, sheet-like pattern, with focal areas displaying fascicular architecture (hematoxylin and eosin stain, 10× magnification).

**Figure 2:**
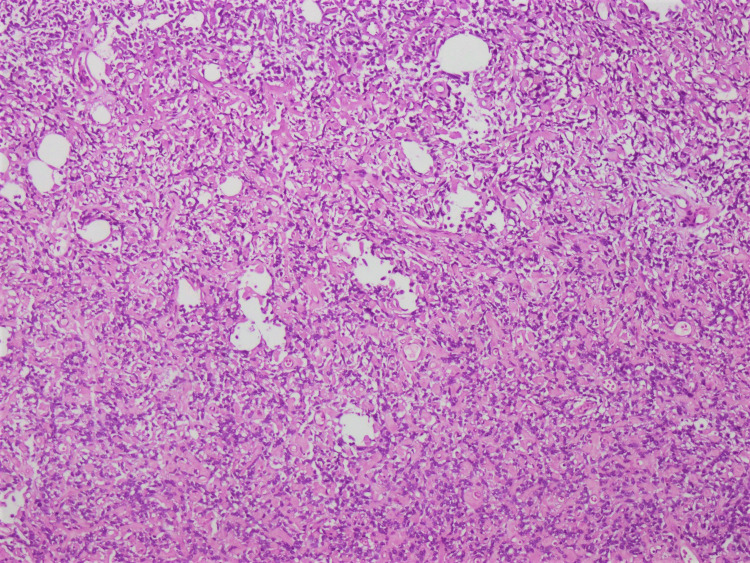
The tumor shows high cellularity, and intervening staghorn-shaped, hemangiopericytoma-like vasculature is noted, set within a hyalinized stroma (hematoxylin and eosin stain, 20× magnification).

**Figure 3:**
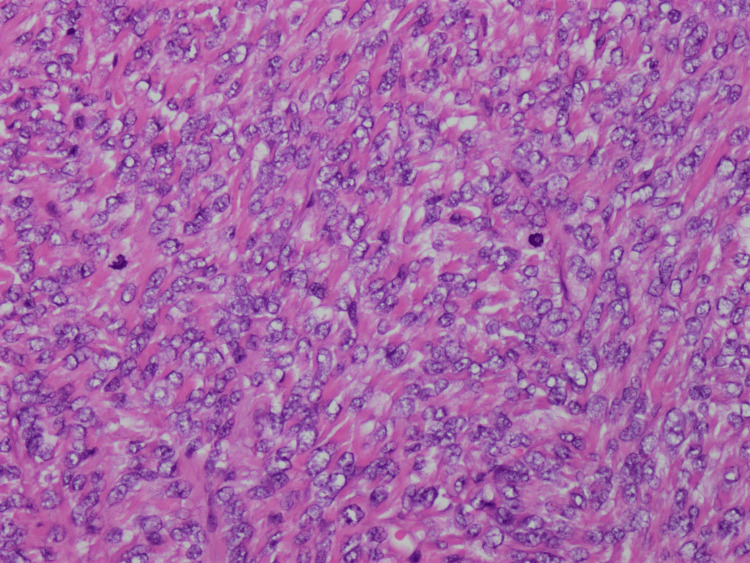
The tumor cells exhibit indistinct cytoplasmic borders, moderate atypia, and scattered mitotic figures (hematoxylin and eosin stain, 40× magnification).

On gross examination, the specimen consisted of multiple prostatic chips measuring 4.1 × 3.8 cm in aggregate and weighing 15 grams. On histological examination, a tumor was identified in all chips. It was composed of spindle-shaped cells arranged in a haphazard, sheet-like pattern, with focal areas displaying fascicular architecture. The cells exhibited indistinct cytoplasmic borders, and nuclei were predominantly uniform, although foci of moderate atypia were observed. Overall, high cellularity was evident. Up to 8 mitotic figures per 10 high-power fields (HPF) were identified, but necrosis and lymphovascular invasion were absent. Intervening staghorn-shaped, or hemangiopericytoma-like, vasculature was evident, and dystrophic calcifications were also noted within a hyalinized stroma. At the periphery, the tumor showed expansile growth and compressed, but did not infiltrate, adjacent benign prostatic glands or overlying urothelium (Figures 1-3). No malignant epithelial component was seen in the tumor.

**Figure 4:**
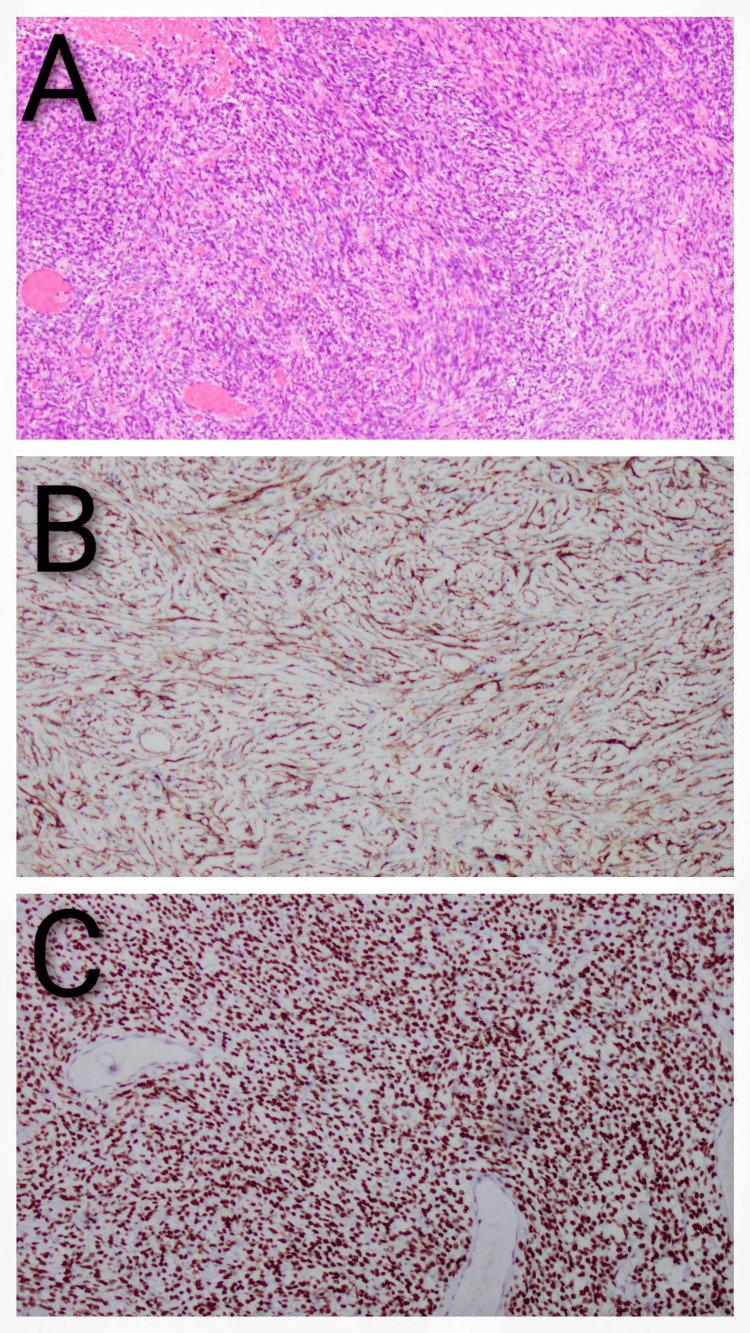
A) Prostatic solitary fibrous tumor (hematoxylin and eosin stain, 20× magnification); B) the tumor shows diffuse membranous positivity (CD34 immunostain, 20× magnification); C) the tumor shows positive nuclear expression (STAT6 immunostain, 20× magnification).

Immunohistochemical analysis demonstrated strong nuclear expression of STAT6 and diffuse CD34 positivity in the neoplastic cells. The tumor cells were negative for epithelial markers (keratin AE1/AE3), myoid or myofibroblastic markers (ASMA, desmin, h-Caldesmon), and neural markers (S100), as well as for the synovial sarcoma marker (SS18) (Figures 4A-4C).

In view of the histomorphological and immunohistochemical findings, a diagnosis of SFT of the prostate was established.

## Discussion

SFT of the prostate is an exceedingly rare mesenchymal neoplasm, posing significant diagnostic challenges due to its histomorphological overlap with other spindle cell lesions. Accurate identification is crucial, as management and prognosis vary across the spindle cell spectrum.

Zhang et al., in their recent systematic review including 40 studies and 74 cases of prostatic SFTs, reported patient ages ranging from 21 to 89 years (mean 57.5 years and median 61.5 years) [4], while Peng et al., in their comprehensive literature review of 25 cases, documented a mean age of 54.2 years. The patient in our report was comparatively older [7].

Histopathological evaluation of prostatic spindle cell lesions can be challenging due to morphological overlap with other entities. Assessment of the NAB2::STAT6 chimeric gene fusion, and subsequent STAT6 expression, has proven to be highly sensitive and specific for diagnosis [8,9]. In a recent systematic review, Zhang et al. [4] reported STAT6 positivity in 97.1% (34/35) of cases, and CD34 positivity in 96.4% (53/55) of cases, highlighting CD34 as another reliable diagnostic marker. BCL‑2 expression was observed in 100% (31/31) of tested cases, further supporting the diagnosis of SFT. In the present case, strong nuclear STAT6 expression and diffuse CD34 immunoreactivity, together with characteristic histological features, established the diagnosis without the need for molecular confirmation.

As highlighted by Liu et al, the presence of even a single adverse feature, such as large tumor size (>10 cm), increased mitotic activity (>4 mitoses per 10 HPFs), high cellularity with nuclear crowding, overlap and pleomorphism, infiltrative margins, necrosis, or hemorrhage should be regarded as indicative of malignant potential and aggressive behavior [10]. In the present patient, high cellularity, moderate nuclear atypia, and 8 mitoses per 10 HPFs suggested a risk for aggressive behavior and malignant SFT. Strong p53 immunoreactivity has been associated with malignant SFT and poor prognosis, whereas benign tumors typically lack such expression [11], p53 staining was not performed in this case, which represents a limitation.

Long-term follow-up is crucial for assessing biological behavior, but our patient did not return to our institution after diagnosis, which remains a limitation. Yilmaz et al. reported a local recurrence rate of 11.6% for prostatic SFT [5], and metastatic cases consistently exhibited at least one adverse histopathological feature, including increased mitotic activity, necrosis, and hypercellularity [12,13]. Tumors demonstrating these features may require radical prostatectomy or wide excision with negative margins, followed by vigilant surveillance, as emphasized by Xu et al. [2].

## Conclusions

This case report contributes to the limited literature on prostatic SFTs and highlights the diagnostic importance of recognizing characteristic histomorphological features, in conjunction with STAT6 and CD34 immunohistochemistry. Although rare, SFT should be considered in the differential diagnosis of spindle cell tumors of the prostate, with the appropriate immunohistochemical panel included to prevent misdiagnosis and inappropriate treatment.
